# Microfluidic cell sorting: Towards improved biocompatibility of extracorporeal lung assist devices

**DOI:** 10.1038/s41598-018-25977-6

**Published:** 2018-05-23

**Authors:** Christian Bleilevens, Jonas Lölsberg, Arne Cinar, Maren Knoben, Oliver Grottke, Rolf Rossaint, Matthias Wessling

**Affiliations:** 1 Department of Anaesthesiology, University Hospital RWTH Aachen University, Aachen, Germany; 2 DWI - Leibniz Institute for Interactive Materials, Aachen, Germany; 3 AVT – Chemical Process Engineering, RWTH Aachen University, Aachen, Germany

## Abstract

Extracorporeal lung assist technology is one of the last options in critical care medicine to treat patients suffering from severe oxygenation and decarboxylation disorders. Platelet activation along with the consequent thrombus formation is a potentially life-threatening complication of this technique. To avoid platelet-dependent clot formation, this study aims at developing a microfluidic cell sorting chip that can bypass platelets prior to the membrane oxygenator of the extracorporeal lung assist device. The cell sorting chips were produced by maskless dip-in laser lithography, followed by soft lithography replication using PDMS. Citrated porcine whole blood with a clinically relevant haematocrit of 17% was used for the cell sorting experiments involving three different blood flow rates. The joint effects of flow focusing and hydrodynamic lifting forces within the cell sorting chip resulted in a reduction of up to 57% of the baseline platelet count. This cell sorting strategy is suitable for the continuous and label-free separation of red blood cells and platelets and is potentially applicable for increasing the biocompatibility and lifetime of current extracorporeal lung assist devices.

## Introduction

Extracorporeal lung assist (ECLA) is a life-saving measure for patients suffering from severe oxygenation or decarboxylation disorders. Acute respiratory distress syndrome (ARDS) in neonates, children and adults is a common indication for ECLA therapy^1–3^. Recent ECLA devices (membrane oxygenators) consist of a hard plastic body containing hollow fibre membranes, which enable gas exchange by solution and diffusion between the patient’s blood and the air flowing through the oxygenator. Despite several attempts to minimize coagulation in the ECLA system, including nitric oxide-releasing membranes, which should reduce platelet adhesion and activation^4^, or alternative anticoagulants such as bivalirudin^5^, the artificial surfaces still cause activation of the coagulation cascade, limiting the use of ECLA to short-term applications for a maximum of a few weeks. Thrombus formation on the membrane is still one of the most critical events^6^. In addition to surface modulations and modification of the systemic anticoagulation strategies, no further or generally new approach has yet been developed to increase the biocompatibility of ECLA devices. Up to 4.5 m^2^ of hollow fibre membrane area contacts the patient’s blood, leading to contact activation of the coagulation system.

Platelets (PLTs) play a crucial role in coagulation and inflammation activation. Activated PLTs initiate the intrinsic and extrinsic coagulation pathways and stimulate the inflammatory pathway by releasing pro-coagulatory and pro-inflammatory mediators from their internal granules^7–9^. Finally, PLTs terminate the coagulation cascade by connecting the growing fibrin network on the oxygenator membrane to a stable clot. Preventing or limiting the contact between PLTs and the large foreign surface of ECLA devices would be a breakthrough in terms of avoiding or at least limiting thrombus formation and the resulting clinical complications.

Microfluidic cell sorting, as described in our study, represents an innovative approach towards improved biocompatibility of ECLA devices, enabling a continuous, passive and label-free separation of PLTs from whole blood with a clinically relevant haematocrit (HCT) of ~17%. The separation principle of the cell sorting chip is based on the joint effects of flow focusing and hydrodynamic lifting forces^10^, leading to an enriched red blood cell (RBC) fraction along the flow path, which is ultimately separated by flow splitting. Experiments with current microfluidic devices using flow focusing are limited to extremely diluted blood samples, with haematocrit values of <1%.

Therefore, this study aims first at the development of a microfluidic cell sorting device that could potentially act as a bypass for PLTs prior to an ECLA device to limit the contact between PLTs and the large oxygenator membrane. Second, experiments should demonstrate the technical feasibility with a clinically relevant haematocrit.

## Materials and Methods

All fabrication steps and analytical methods performed in this study were in accordance with the relevant guidelines and regulations.

### Fabrication of microfluidic cell sorting chips

The cell sorting chip was prepared in two steps^11^ with the maskless dip-in laser lithography method^12–15^ to produce the microfluidic master, a negative structure of the flow channel, which was replicated by the soft lithography method^16^. The master was printed with a Photonic Professional GT system (Nanoscribe GmbH; Karlsruhe, Germany) on a silanized^17^ microscopy glass substrate (25 × 75 × 1 mm, Sigma Aldrich; Munich, Germany) by means of focusing (25 × NA = 0.8) a near infrared laser beam into a droplet of photoresist (IP-L, Nanoscribe GmbH; Karlsruhe, Germany) to initiate two-photon polymerization of the negative master. The master was printed in sections with hexagonal splitting, applying slicing and hatching distances of 600 nm and 200 nm, respectively. The sidewalls of the master were printed with a thickness of 2 µm, resulting in a polymerized shell and a supported liquid core. The printed master was developed for 10 min in propylene glycol methyl ether acetate (PGMEA) (≥99.5%, Sigma Aldrich; Munich, Germany) and rinsed for 3 min with isopropanol (≥99.8% (GC), Sigma Aldrich; Munich, Germany). The inner core was polymerized under UV light exposure for 12 h. From the resulting master, the cell sorting chips were replicated by moulding poly(dimethylsiloxane) (PDMS) (Sylgard® 184 plus curing agent, 10:1 (w/w), Dow Corning; Wiesbaden, Germany) onto the master, which was further cross-linked overnight at 60 °C in an oven. The cured PDMS mould was cut with a scalpel, and holes for the connecting tubing (fine bore polythene, inner diameter: 0.38 mm, outer diameter: 1.09 mm, Smiths Medical; Minneapolis, USA) were punched into the PDMS slab using a biopsy puncher. The PDMS slab was plasma bonded to a microscopy glass substrate (25 × 75 × 1 mm, Sigma Aldrich; Munich, Germany) under 100 Pa oxygen pressure at 40 W for 30 s (TePla 100 Plasma System, PVA MPS GmbH; Wettenberg, Germany)^18^. In a final step, 20 cm tubes with connected Luer Lock syringe needles were glued (Max Repair Extreme, UHU; Bühl/Baden, Germany) to the inlets and outlets of the cell sorting chip.

### Blood collection from healthy pigs

For each experiment, we received 1–2 mL of a fresh, citrated blood sample from healthy male domestic pigs (Governmental Animal and Use Committee of North Rhine-Westphalia Approval No. 84 02.04.2015.A497). After withdrawal, the blood was stored at room temperature for a maximum of 60 min on a sample roller and analysed by a cell counter shortly before the experiments (Celltac α MEK 6550, Nihon Kohden Inc.; Rosbach, Germany). To achieve comparable conditions to those in previous published *in vivo* ECLA studies^19–25^, porcine blood samples were diluted to a HCT value of 17% and subsequently assigned to one of the experimental groups in a randomized manner.

### Experimental groups

The experiments were performed in three groups with variable proportions of blood and sheath fluid (isotonic 0.9% NaCl solution). The flow rate equalled 40 µL h^−1^ blood and 110 µL h^−1^ NaCl in the 1st group (LOW), 60 µL h^−1^ blood and 90 µL h^−1^ NaCl in the 2nd group (MID) and 80 µL h^−1^ blood and 70 µL h^−1^ NaCl in the 3rd group (HIGH).

### Cell sorting experiments

Prior to each experiment, the channels of the cell sorting chip were focused at 10× magnification (EVOS FL Auto; Thermo Fisher Scientific Inc.; Darmstadt, Germany), wetted with isopropanol, and then flushed with a NaCl-trisodium citrate mixture (1:10, S-Monovette; Sarstedt Inc.; Nürmbrecht, Germany), both at a flow rate of 1 mL min^−1^ for 10 min. Prior to the experiment, the blood inlet was connected via tubes to a 1 mL gas-tight syringe (SGE Analytical Science; Meerbusch, Germany) filled with diluted porcine blood. The syringe was mounted on a syringe pump (PHD Ultra, Harvard Apparatus/Hugo Sachs; March, Germany), and the flow rate was set according to the randomized experimental groups (40, 60, or 80 µL h ^−1^). The sheath inlet was connected via tubes to an identical 1 mL gas-tight syringe filled with a 1:10 mixture of NaCl and trisodium citrate, and the flow rate of the second identical syringe pump was set according to the experimental group (110, 90 or 70 µL h^−1^). The RBC outlet of the chip was connected via tubes to a syringe pump, which was set to a withdrawal rate of 109 µL h^−1^ in accordance with the channel geometry. This condition was essential to fix the degrees of freedom and stabilize the flow distribution with equal flow splitting at the outlet. Blood from the PLT outlet of the chip was collected without withdrawal. The experiments were performed, until both outlet tubes were filled with blood, corresponding to approximately 23 µL.

### Blood sampling and analysis

As soon as both the outlet tubes were completely filled with blood, the blood was transferred to 1.5 mL standard reaction tubes. The inlet tube was processed in the same manner. The blood analysis was performed in duplicate using the predilution mode of the blood cell counter (10 µL blood +200 µL diluent). The RBC/PLT ratio and the RBC/white blood cell (WBC) ratio were calculated from the cell counts at the outlets and normalized to the cell counts at the inlet, according to the following formulae:

$${\bf{RBC}}/{\bf{PLT}}=\frac{{\bf{RB}}{{\bf{C}}}_{{\bf{out}}}/{\bf{PL}}{{\bf{T}}}_{{\bf{out}}}}{{\bf{RBC}}{C}_{{\bf{in}}}/{\bf{PL}}{{\bf{T}}}_{{\bf{in}}}}$$

$${\bf{RBC}}/{\bf{WBC}}=\frac{{\bf{RB}}{{\bf{C}}}_{{\bf{out}}}/{\bf{WB}}{{\bf{C}}}_{{\bf{out}}}}{{\bf{RB}}{{\bf{C}}}_{{\bf{in}}}/{\bf{WB}}{{\bf{C}}}_{{\bf{in}}}}$$

### Evaluation of the cell sorting principle

To prove the cell sorting principle of the chip and ensure that the measurements were related to the joint effects of flow focusing and hydrodynamic lifting within the cell sorting chip, the maximal height of the blood cells along the flow path was measured. RBCs should reach an adequate separation height from the PLTs before the final separation step of splitting the channel into an ECLA outlet and a bypass outlet. For this purpose, six measurement points (0.5, 2, 5, 9.5, 15, and 19.5 mm) were added to the cell sorting chips along the 19.5 mm channel to determine the maximal cell height within the chip. Six repeated measures were performed for each of the six measurement points for the three experimental groups (108 data points). Images from the EVOS microscope were saved and used for the determination of the maximum RBCs height, using ImageJ (ImageJ 1.46, National Institutes of Health; Bethesda, USA).

### Detection of potential clot formation sites using pro-coagulatory blood within the chip

To identify potential clot formation sites within the cell sorting chip, we performed one exemplary experiment with heparinized porcine blood, with the addition of 5 international units (IU mL^−1^) heparin (5.000 IU mL^−1^, Rotexmedica; Trittau, Germany) instead of citrated blood. The sample was mixed with a fluorescent dye (3.3′-dihexyloxacarbocyanine iodide/DiOC6, #318426, Sigma Aldrich; Munich, Germany) at a final concentration of 0.5 µg mL^−1^ and incubated at room temperature for 10 min in the dark. This staining is specific for thrombus formation and PLT aggregate visualization^26^. Prior to the connection of the blood sample to the microfluidic chip, we antagonized the heparin with 5 IU mL^−1^ protamine (1.000 IU mL^−1^, MEDA Pharma, Cologne, Germany) to enforce clot formation. The experiment was performed using the flow settings of the HIGH group (80 µL h^−1^ blood, 70 µL h^−1^ NaCl). Images of the inlet and the outlet of the channel were taken at 5, 30, and 45 min after the experiments started, using the green fluorescent protein (GFP) filter (AMEP4651) of the EVOS microscope, to visualize the absorption maximum of DiOC6 at 485 nm.

### Experiments with an increased flow rate

The cell sorting efficacy was evaluated by exemplary experiments using increased flow rates with varying proportions of blood and sheath fluid at 400/800 µL h^−1^ and 600/1200 µL h^−1^, respectively, with two measurements each, based on the described experimental settings.

### Statistical analysis

Data are presented as the mean ± standard deviation (SD). The RBC/PLT and RBC/WBC ratios at the outlets (ECLA and bypass) of the cell sorting chip, with normalization to the values at the inlet, were checked for a Gaussian distribution, and a parametric, unpaired, two-tailed t-test was performed. The results were regarded as significantly different if the calculated p value was <0.05. All statistical analyses and the graph design were performed using GraphPad Prism software (Prism® 6.04, GraphPad Software; California, US).

### Data availability statement

The corresponding author declares that all raw data material is available at the DWI - Leibniz Institute for Interactive Materials and the Dept. of Anaesthesiology of the University Hospital in Aachen, Germany.

## Results

### Design of the microfluidic cell sorting chip

**Figure 1:**
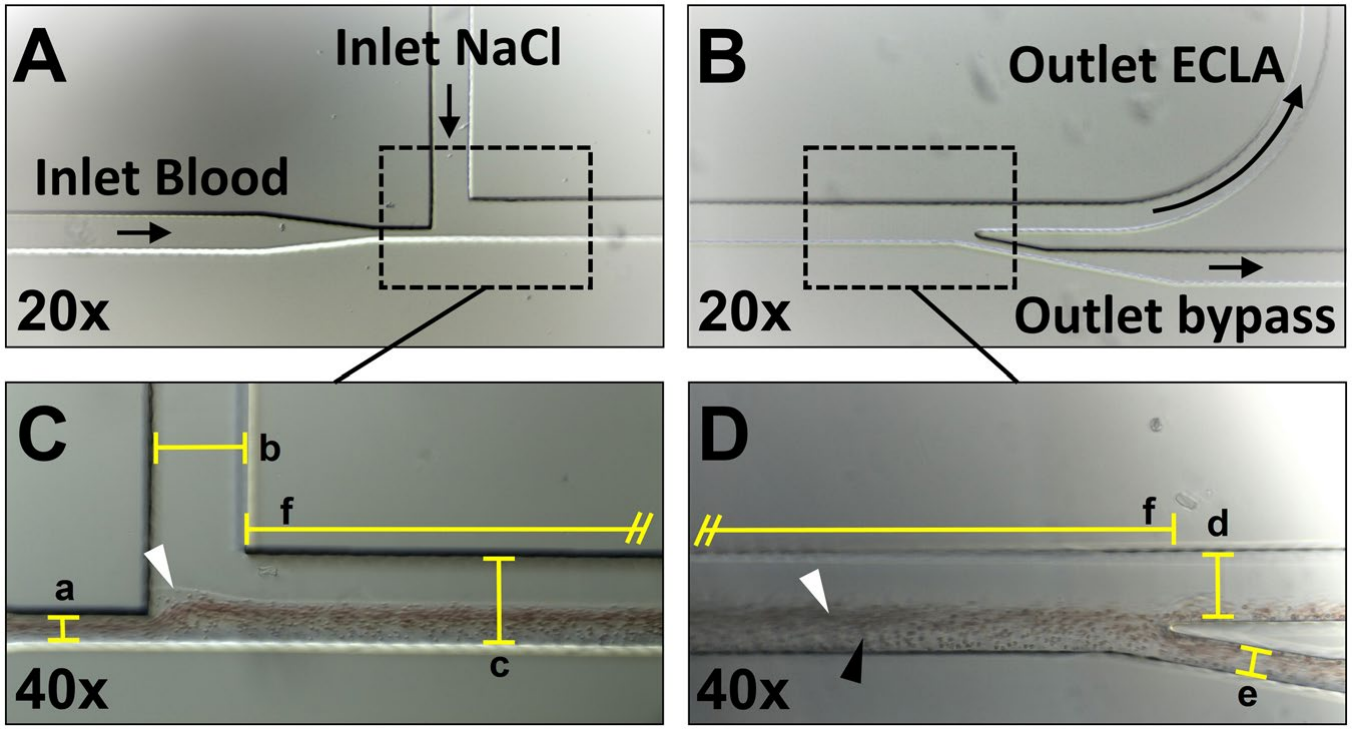
The microfluidic channel consists of an inlet for blood and NaCl (**A**) and an assigned ECLA and bypass outlet (**B**). Citrated blood enters the cell sorting chip and is focused by NaCl at the inlet (**C**/white marker). RBCs are lifted into the upper regions of the channel (**D**/white marker), and PLTs remain in the lower parts (**D**/black marker). Nominal chip dimensions: a = 30 µm, b = 110 µm, c = 110 µm, d = 80 µm, e = 30 µm, and f = 19.5 mm.

The design of the microfluidic cell sorting chip is inspired by the work of T.M. Geislinger *et al*. integrating two inlet and two outlet channels connected via a separation channel^10,27^. All channels are fabricated with a constant thickness of 100 µm. Citrated whole blood enters the cell sorting chip through a narrow inlet (a = 30 µm) and is focused by the NaCl sheath flow (b = 110 µm) entering the separation channel perpendicular to the blood flow. In the separation channel (c = 110 µm, f = 19.5 mm), the blood cells are lifted from the lower channel wall and start to separate due to their different sizes and shapes. The final separation of red blood cells (RBCs), white blood cells (WBCs) and platelets (PLTs) occurs at the outlet, where the flow splits into two fractions assigned to the extracorporeal lung assist (ECLA) (d = 80 µm) and bypass (e = 30 µm) outlets (Fig. 1).

### Cell sorting efficacy for platelets (PLTs)

**Figure 2:**
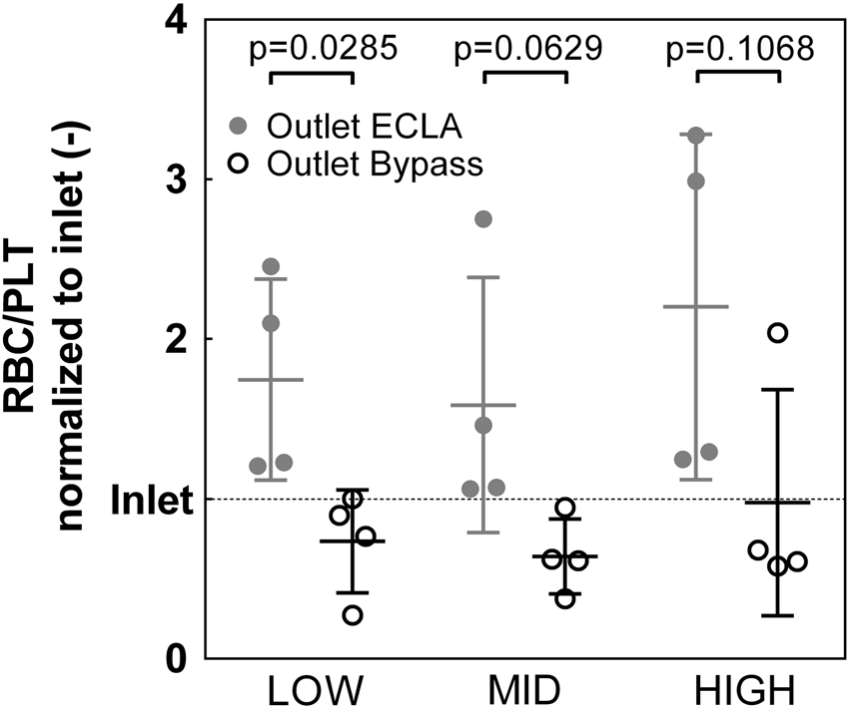
Fraction of red blood cells (RBCs) and platelets (PLTs) at the assigned extracorporeal lung assist (ECLA) outlet (grey circle) and the assigned bypass outlet (black hollow circle) normalized to the fraction of the inlet (dotted line). The PLT fraction in the ECLA outlet is significantly reduced in the LOW group (40 µL h^−1^ blood + 110 µL h^−1^ NaCl, p < 0.05). In the MID group (60 µL h^−1^ blood + 90 µL h^−1^ NaCl) and the HIGH (80 µL h^−1^ blood + 70 µL h^−1^ NaCl) group, the reduction in the PLT fraction in the ECLA outlet was not significant.

In all experimental groups, fewer PLTs were in the assigned ECLA outlet of the device than in the assigned bypass outlet. Within the LOW group, a significant reduction of 57.9% in the initial PLT fraction was measured in the assigned ECLA outlet compared to the bypass outlet (RBC/PLT: 0.74 ± 0.32 vs. 1.75 ± 0.63, p = 0.0285). For the MID group (RBC/PLT: 0.64 ± 0.23 vs. 1.59 ± 0.80, p = 0.0629) and the HIGH group (RBC/PLT: 0.98 ± 0.71 vs. 2.20 ± 1.08, p = 0.1068), a clear tendency towards a reduced PLT fraction in the assigned ECLA outlet was measured (MID: 59.7%, HIGH: 55.6%), but the separation efficacy failed to reach significance (Fig. 2).

### Cell sorting efficacy for white blood cells (WBCs)

**Figure 3:**
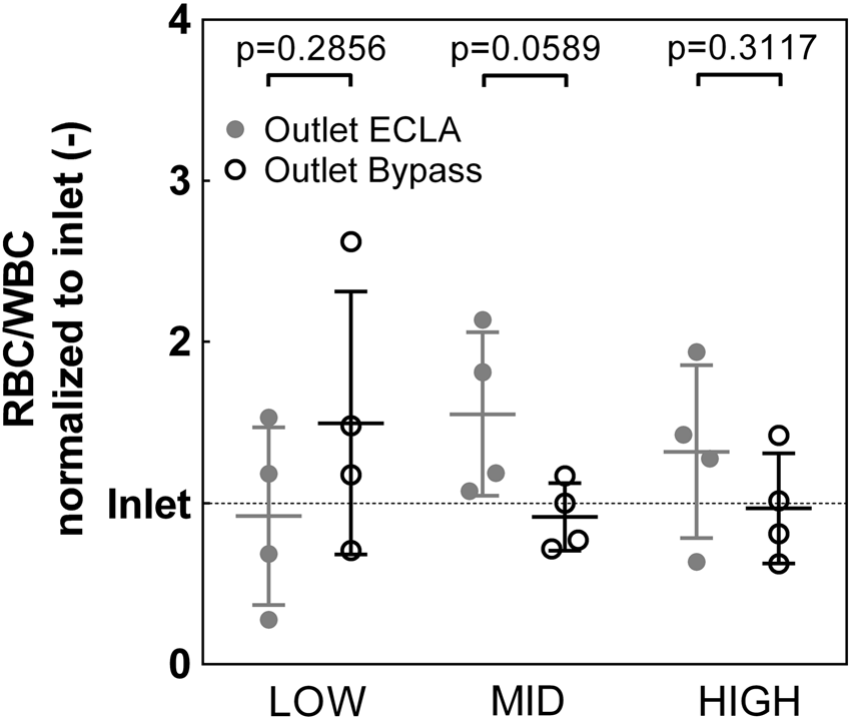
Fraction of red blood cells (RBCs) and white blood cells (WBCs) at the assigned extracorporeal lung assist (ECLA) outlet (grey circle) and the assigned bypass outlet (black hollow circle) normalized to the fraction at the inlet (dotted line). The WBC fraction tends to be higher in the ECLA outlet than in the bypass outlet in the LOW group (40 µL h^−1^ blood + 110 µL h^−1^ NaCl) but tends to be lower in the ECLA outlet than in the bypass outlet in the MID (60 µL h^−1^ blood + 90 µL h^−1^ NaCl) and HIGH (80 µL h^−1^ blood + 70 µL h^−1^ NaCl) groups. The differences did not reach significance (p > 0.05).

The cell sorting principle for the separation of RBCs and WBCs did not yield clear results in comparison to the RBC/PLT separation results. In the LOW group, the WBC fraction was 62% higher in the assigned ECLA outlet than in the assigned bypass outlet, whereas in the MID and HIGH groups, the WBC fraction was 41% and 26%, respectively, lower in the assigned ECLA outlet (RBC/WBC: LOW: 1.50 ± 0.82 vs. 0.92 ± 0.55, p = 0.2856; MID: 0.92 ± 0.21 vs. 1.55 ± 0.51, p = 0.0589; HIGH: 0.97 ± 0.34 vs. 1.32 ± 0.53, p = 0.3117) (Fig. 3).

### Cell sorting principle

**Figure 4:**
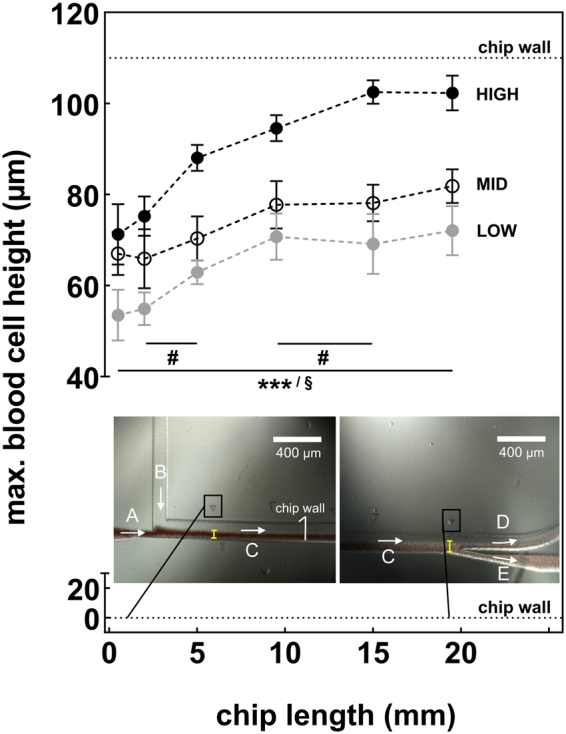
The maximal blood cell height within the cell sorting chip was determined at six measurement points (yellow bar) and changed significantly over the chip length independent of the experimental group (***p < 0.001). From 2–5 mm and from 9.5–15 mm chip length, the blood cell height showed a significantly stronger increase in the HIGH group (^#^p < 0.05). At each of the measurement points, the maximal blood cell height was significantly lower in the LOW group than in the MID and HIGH groups (^§^p < 0.05). (**A**) Citrated blood inlet for each of the experimental groups (40, 60, 80 µL h^−1^). (**B**) Sheath fluid inlet (110, 90, 70 µL h^−1^). (**C**) Flow direction. (**D**) ECLA outlet. (**E**) Bypass.

A significant effect on the changes in blood cell height was measured at different positions in the separation channel, independent of the experimental group (***p = 0.001). This finding proves the correct functionality of the cell sorting principle (hydrodynamic lifting forces). The variable proportions of blood and sheath fluid within the experimental groups had a major impact on the blood cell height and cell sorting efficacy. The measured maximal blood cell height was significantly lower at each of the six measurement points for the LOW group than for the MID and HIGH groups (^§^p = 0.05). The sorting process in the LOW group started with 53.5 ± 5.6 µm vs. MID 67 ± 4.7 µm vs. HIGH 71.3 ± 6.6 µm at the 0.5 mm measurement point and finished with 72.1 ± 5.4 µm vs. MID 81.84 ± 3.7 µm vs. HIGH 102.3 ± 3.8 µm at the 19.5 mm measurement point (Fig. 4).

### Detection of potential clot formation sites using pro-coagulatory blood within the chip

**Figure 5:**
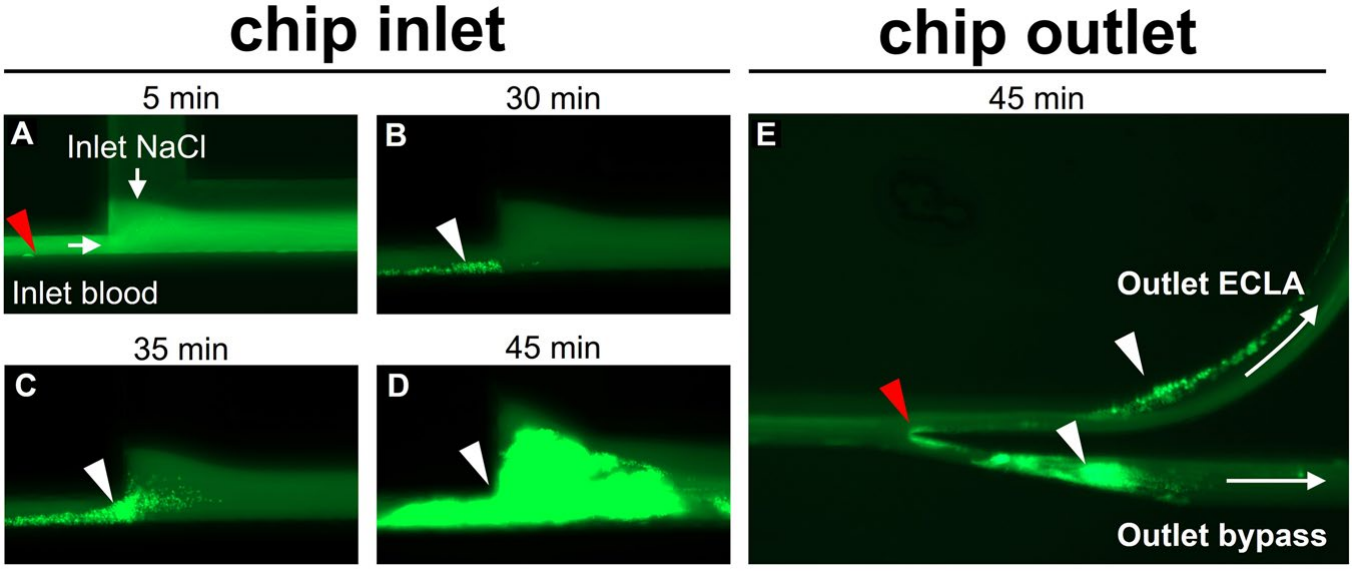
DiOC6 staining of pro-coagulatory porcine blood at the inlet (**A**–**D**) and the outlet (**E**) of the cell sorting chip. The formation of a blood clot over time (white markers) was observed after 5, 30, 35, and 45 min at the inlet and after 45 min at the outlet. Red markers indicate potential start locations for clot formation within the chip.

Blood coagulation was visualized by a fluorescence signal from the DiOC6 dye during a cell sorting experiment with pro-coagulatory porcine blood. Clot formation starts after 5 min at the blood inlet (Fig. 5A/red marker). This position of the channel features the lowest height (30 µm) and thus high shear rates within the cell sorting chip. From this starting position, the clot grows slowly during the first 30 min (Fig. 5B/white marker). The initial growing blood clot decreases the channel cross section while the blood is further supplied by a constant flow rate, leading to higher shear forces accelerating the clot formation notably (Fig. 5C/white marker). Finally, after 45 min, the blood clot blocks the entire inlet (Fig. 3D/white marker). Parts of the blood clot from the inlet attach at the tip, which separates the outlet channels (Fig. 5E/ECLA outlet/Bypass outlet, red marker), and initiate further clot formation at both outlets (Fig. 5E/white markers).

### Effect of increased flow rate

Compared to the experiments with lower flow rates, the exemplary upscaling experiments with up to 10-fold increased blood flow rates did not lead to a separation of RBCs, PLTs and WBCs.

## Discussion

As a new approach for minimized clot formation in ECLA systems, we aimed at the development of a microfluidic cell sorting chip to bypass PLTs. For the rapid fabrication of multiple chip designs with short development cycles, we used a new methodology of maskless dip-in laser lithography in combination with soft lithography replication^11^. These cell sorting chips enable a continuous and label-free separation of blood cells based on the joint effects of flow focusing using a sheath flow and hydrodynamic lifting forces.

We could prove the cell sorting principle of the microfluidic chip, as we measured an increasing blood cell height within the chip, which was also related to the changing blood flow rates in our three experimental groups. The separation process within the channel is consistent and reproducible, showing no clot formation and a significant reduction in the baseline PLT count of up to 57% at the outlet of the cell sorting chip. These results were achieved with diluted and citrated porcine blood in accordance with a clinically relevant HCT value of ~17% for *in vivo* ECLA settings. The chip mainly worked for the LOW sample group, where the degree of cell separation was significant. This result is due to a higher number of blood cells in the MID and HIGH groups, leading to more pronounced particle-particle interactions, which would ultimately result in a statistical distribution for the different cell types^28^. In contrast, in the LOW group, the joint effects of flow focusing and hydrodynamic lifting forces are superior to the particle-particle interaction, and a significant sorting effect is achieved.

Additionally, we could identify potential blood clot formation sites within the cell sorting chip using a thrombus-specific fluorescent dye and porcine blood in a pro-coagulatory state.

From a biological point of view, a PLT reduction of up to 57% within an ECLA device limits the PLT contact activation, which triggers coagulation and inflammation as PLTs release pro-coagulatory and pro-inflammatory mediators from their internal granules upon contact with foreign surfaces^8^ Furthermore, activated platelets express P-selectin on their cell surface, which enables leucocyte rolling and activation by binding to the P-selectin-glycoprotein ligand (PSGL-1) on the surface of circulating leucocytes, followed by further bonds between the cells and the release of neutrophil extracellular traps (NETs)^29^. Additionally, activated PLTs release pro-coagulatory microparticles (MPs) carrying tissue factor as an activator of the extrinsic coagulation system on their surface^30,31^ As a result, a growing fibrinogen biofilm consisting of cell residues (PLTs, WBCs, RBCs, and MPs) settles on the oxygenator membrane of ECLA devices, and finally PLTs connect the fibrin network to a stable blood clot. In summary, the reduction in the number of PLTs contacting an ECLA device may reduce the risk of clot formation on the membrane, the potential for embolic events, and the need for system exchange. System exchange was reported previously to be essential for up to 27% of long-term ECLA patients^6^.

Despite a 57% reduction in PLTs, as detected in the recent *in vitro* study, 60 × 10^3^–120 × 10^3^ PLTs µL^−1^ still contact the foreign surface. The recommendations for clinical PLT transfusions from the latest guidelines declare that 50 × 10^3^–100 × 10^3^ PLTs µL^−1^ is still adequate for sufficient coagulation. Although these guidelines are not necessarily transferable to our *in vitro* setting, one can still argue that the remaining PLT count in our setting is still sufficient for membrane clot formation^31^.

Thus, from a clinical point of view, our cell sorting device did not reduce the PLT counts to a level that is low enough to prevent coagulation sufficiently. Nevertheless, the combination of 57% PLT reduction with well-established heparin-based anticoagulant surface coatings of recent ECLA devices^32–35^ or with alternative anticoagulant coatings such as bivalirudin^5^ might be a promising strategy. The ultimate way to decrease ECLA-related complications should be a combination of several new strategies and not one single modification. For example, Major and colleagues showed that the combination of nitric oxide-releasing surfaces and covalently attached argatroban as a thrombin inhibitor lowers the PLT contact activation better than the application of a single method^36^.

Therefore, from a technical point of view, further improvement and development of recent cell sorting devices seems worthwhile since we could establish an improved cell sorting device, which was operated with a clinically relevant haematocrit value of ~17%, compared to the device in the pioneering study of Geislinger and colleagues^27^. They used a different design and showed cell sorting success with experimental HCT values of 0.1% in a proof-of-principle setting. However, our results show that a complete clearance of PLTs from the ECLA device does not seem feasible with a high haematocrit value. In contrast, Hou and colleagues showed better discrimination between healthy and malaria-infected RBCs in their microfluidic device with increasing HCT. Their study yielded up to 90% separation efficacy in a 40% HCT setting^37^. Hou and colleagues were not interested in the separation of PLTs from RBCs; however, the separation mechanism is based on the same parameters: size, shape and deformability. The fact that clinically relevant HCT values were suitable for the separation process generally proved that the microfluidic technology is suitable for medical applications; however, increasing the throughput of microfluidic devices beyond the use as point-of-care diagnostics remains one of the main challenges^38^.

In clinical routines such as ECLA, an upscaling to higher flow rates by parallelization of multiple channels is necessary. Starting with *in vivo* ECLA mouse models, a 5 mL min^−1^ blood flow would be sufficient, which would already necessitate an upscaling factor of ~3.7 k, as we performed the experiments with a maximum of 80 µL h^−1^. For sufficient extracorporeal CO_2_ removal from an adult patient, a minimal flow of 6 mL kg^−1^ bodyweight is recommended^39^. This value equals 480 mL min^−1^ for an 80 kg patient, requiring an upscaling factor of at least ~355 k.

Another challenge results from the dilution with sheath fluid, which needs to be removed from the circuit by dialysis after blood cell separation^40^. Different architectures of microfluidic cell sorting devices might be an alternative, especially those that work without flow focusing by a sheath fluid^41–49^. A potential approach is dean flow fractionation within a spiral microchannel combining shear- and wall-induced lifting forces. Two rotating vortices force cells of different size and shape into opposite regions of the channel^50^.

In contrast to microfluidic techniques for diagnostic use, most clinical applications depend on physiological flow conditions, which necessitate a scale-up of current microfluidic devices. By means of parallelization of a single optimized separation channel, the transition from diagnostic use to the clinical application seems possible and the most promising strategy. With respect to the need for physiological flow conditions (moderate shear forces) for later *in vivo* applications, the maximal flow rate within a single sorting channel is limited to prevent haemolysis.

Thompson and colleagues recently described a microfluidic artificial lung that enables oxygenation and decarboxylation with a maximal blood flow rate of 0.5 mL min^−1^, which means that 960 devices are still needed for sufficient CO_2_ removal in an adult patient^51^. As stated by Sackmann and colleagues, microfluidic tools definitely have the potential to make substantial contributions to biology and medical research^28^. However, it remains challenging to use microfluidics for medical devices rather that a diagnostic tool.

### Limitations of the study

The major limitations in this experimental setting are the low flow rates (µl h^−1^) of this device, which creates a large scale-up and the resulting need for dialysis of the sheath fluid from the system after cell sorting. For further analysis of the cell sorting effect on coagulation, inflammation and haemolysis, higher flow rates for adequate blood sample volumes are essential. Furthermore, the recent experiments were performed at room temperature because the project was the first experimental trial, and we used porcine blood to evaluate the technique. Scale-up studies should be performed in a setting using human blood at 37 °C as the physiological temperature will influence the coagulation and blood flow properties within the device. In terms of translational medicine, a dataset describing the cell sorting efficacy under physiological and scale-up conditions in porcine blood is important for the establishment of follow-up studies of porcine ECLA models for realistic testing of the device *in vivo*.

## Conclusion

We showed that microfluidic cell sorting based on the joint effects of flow focusing and hydrodynamic lifting forces is a suitable approach for continuous and label-free separation of red blood cells and platelets in whole blood with a clinically relevant haematocrit. Thus, from a biological point of view, we conclude that a platelet reduction of up to 57% in extracorporeal lung assist devices will be beneficial for patients, although from a clinical point of view, the remaining platelets might still be sufficient for blood clot formation within the devices. However, from the technical point of view, a microfluidic cell sorting approach appears to be a realistic strategy for bypassing platelets and limiting the risk of thrombus formation on the oxygenator membrane.

## References

[1] Rambaud J, Guilbert J, Guellec I, Renolleau S (2012). A pilot study comparing two polymethylpentene extracorporeal membrane oxygenators. Perfusion..

[2] Hirshberg E, Miller RR, Morris AH (2012). Extracorporeal membrane oxygenation in adults with acute respiratory distress syndrome. Curr Opin Crit Care..

[3] Nolan H, Wang D, Zwischenberger JB (2011). Artificial lung basics: fundamental challenges, alternative designs and future innovations. Organogenesis..

[4] Amoako KA (2013). Fabrication and *in vivo* thrombogenicity testing of nitric oxide generating artificial lungs. J Biomed Mater Res A..

[5] Pieri M (2013). Bivalirudin versus heparin as an anticoagulant during extracorporeal membrane oxygenation: a case-control study. J Cardiothorac Vasc Anesth..

[6] Dornia C (2014). Analysis of thrombotic deposits in extracorporeal membrane oxygenators by multidetector computed tomography. ASAIO J..

[7] Schmaier AH (2016). The contact activation and kallikrein/kinin systems: pathophysiologic and physiologic activities. J Thromb Haemost..

[8] Rossaint J, Zarbock A (2015). Platelets in leucocyte recruitment and function. Cardiovasc Res..

[9] Jenne CN, Urrutia R, Kubes P (2013). Platelets: bridging hemostasis, inflammation, and immunity. Int J Lab Hematol..

[10] Geislinger TM, Franke T (2014). Hydrodynamic lift of vesicles and red blood cells in flow–from Fåhræus & Lindqvist to microfluidic cell sorting. Adv Colloid Interface Sci..

[11] Lölsberg, J. *et al*. 3D Nanofabrication inside rapid prototyped microfluidic channels showcased by wet-spinning of single micrometre fibres. *Lab Chip*. **18**, 1341–1348 (2018). 10.1039/c7lc01366c 29619449

[12] Strickler, J. H. & Webb, W. W. Two-photon excitation in laser scanning fluorescence microscopy. *Proc*. **90**, 10.1117/12.47787 (1991).

[13] Maruo S, Nakamura O, Kawata S (1997). Three-dimensional microfabrication with two-photon-absorbed photopolymerization. Opt Lett..

[14] Kawata S, Sun HB, Tanaka T, Takada K (2001). Finer features for functional microdevices. Nature..

[15] Sun H-B-, Kawata S (2004). Two-Photon Photopolymerization and 3D Lithographic Microfabrication. Advances in polymer sciences..

[16] Xia Y, Whitesides GM (1998). Soft Lithography. Annu. Rev. Mater. Sci..

[17] Kash, L. Mittal. “Silanes and Other Coupling Agents, Volume 4.” CRC Press. (2007).

[18] Bhattacharya S, Datta A, Berg JM, Gangopadhyay S (2005). Studies on surface wettability of poly(dimethyl) siloxane (PDMS) and glass under oxygen-plasma treatment and correlation with bond strength. Journal of Microelectromechanical Systems..

[19] Kopp R, Kuhlen R, Max M, Rossaint R (2003). Evidence-based medicine of the acute respiratory distress syndrome. Anaesthesist..

[20] Kopp R (2010). Hemocompatibility of a miniaturized extracorporeal membrane oxygenation and a pumpless interventional lung assist in experimental lung injury. Artif Organs..

[21] Kopp R (2011). A miniaturized extracorporeal membrane oxygenator with integrated rotary blood pump: preclinical *in vivo* testing. ASAIO J..

[22] Kajimoto M (2015). Differential effects of octanoate and heptanoate on myocardial metabolism during extracorporeal membrane oxygenation in an infant swine model. Am J Physiol Heart Circ Physiol..

[23] Ledee DR (2015). Pyruvate modifies metabolic flux and nutrient sensing during extracorporeal membrane oxygenation in an immature swine model. Am J Physiol Heart Circ Physiol..

[24] Stang K (2015). First *In Vivo* Results of a Novel Pediatric Oxygenator with an Integrated Pulsatile Pump. ASAIO J..

[25] Itoh H (2016). Effect of the Pulsatile Extracorporeal Membrane Oxygenation on Hemodynamic Energy and Systemic Microcirculation in a Piglet Model of Acute Cardiac Failure. Artif Organs..

[26] Gibbins, J. M. & Mahaut-Smith, M. P. “*Platelets and Megakaryocytes*.” Vol. 272 (2004).

[27] Geislinger, T. M., Eggart, B., Braunmüller, S., Schmid, L. & Franke, T. Separation of blood cells using hydrodynamic lift. *Appl. Phys.Lett*. **100**, 10.1063/1.4709614 (2012)

[28] Jacob, N. Israelachvili. Intermolecular and Surface Forces. *Academic Press*. 3rd edition (2011).

[29] Rossaint J (2014). Synchronized integrin engagement and chemokine activation is crucial in neutrophil extracellular trap-mediated sterile inflammation. Blood..

[30] Mooberry MJ (2016). Procoagulant microparticles promote coagulation in a factor XI-dependent manner in human endotoxemia. J Thromb Haemost..

[31] Estcourt LJ (2017). British Committee for Standards in Haematology. Guidelines for the use of platelet transfusions. Br J Haematol..

[32] Videm V, Fosse E, Mollnes TE, Garred P (1988). Complement activation by extracorporeal circulation: effects of precoating a membrane oxygenator circuit with human whole blood. Scand J Thorac Cardiovasc Surg..

[33] Gunaydin S (2002). Clinical performance and biocompatibility of poly(2-methoxyethylacrylate)-coated extracorporeal circuits. Ann Thorac Surg..

[34] Kopp R, Mottaghy K, Kirschfink M (2002). Mechanism of complement activation during extracorporeal blood-biomaterial interaction: effects of heparin coated and uncoated surfaces. ASAIO J..

[35] Gunaydin S, McCusker K, Sari T, Onur MA, Zorlutuna Y (2010). Clinical performance and biocompatibility of hyaluronan-based heparin-bonded extracorporeal circuits in different risk cohorts. Interact Cardiovasc Thorac Surg..

[36] Major TC (2014). The effect of a polyurethane coating incorporating both a thrombin inhibitor and nitric oxide on hemocompatibility in extracorporeal circulation. Biomaterials..

[37] Hou HW (2010). Deformability based cell margination - a simple microfluidic design for malaria-infected erythrocyte separation. Lab Chip..

[38] Sua W, Gaoab X, Jianga L, Qin J (2015). Microfluidic platform towards point-of-care diagnostics in infectious diseases. Journal of Chromatography A..

[39] Bein T (2013). Lower tidal volume strategy (∼3 ml/kg) combined with extracorporeal CO2 removal versus ‘conventional’ protective ventilation (6 ml/kg) in severe ARDS: the prospective randomized Xtravent-study. Intensive Care Med..

[40] de Jong J, Lammertink RG, Wessling M (2006). Membranes and microfluidics: a review. Lab Chip..

[41] Di Carlo D, Irimia D, Tompkins RG, Toner M (2007). Continuous inertial focusing, ordering, and separation of particles in microchannels. PNAS..

[42] Davis JA (2006). Deterministic hydrodynamics: Taking blood apart. PNAS..

[43] Warkiani ME, Tay AK, Guan G, Han J (2015). Membrane-less microfiltration using inertial microfluidics. Sci Rep..

[44] Henry E (2016). Sorting cells by their dynamical properties. Sci Rep..

[45] Rafeie M, Zhang J, Asadnia M, Li W, Warkiani ME (2016). Multiplexing slanted spiral microchannels for ultra-fast blood plasma separation. Lab Chip..

[46] Guo Q., Duffy S. P., Matthews K., Islamzada E. & Ma, H. Deformability based Cell Sorting using Microfluidic Ratchets Enabling Phenotypic Separation of Leukocytes Directly from Whole Blood. *Sci Rep*. **26**, 10.1038/s41598-017-06865-x (2017). 10.1038/s41598-017-06865-x PMC5529452 28747668

[47] Vernekar R (2017). Anisotropic permeability in deterministic lateral displacement arrays. Lab Chip..

[48] Ranjan S, Zeming KK, Jureen R, Fisher D, Zhang Y (2014). DLD pillar shape design for efficient separation of spherical and non-spherical bioparticles. Lab Chip..

[49] Huang LR, Cox EC, Austin RH, Sturm JC (2004). Continuous particle separation through deterministic lateral displacement. Science..

[50] Thompson, A. J. *et al*. A small-scale, rolled-membrane microfluidic artificial lung designed towards future large area manufacturing. *Biomicrofluidics*. **11**, 10.1063/1.4979676, (2017). 10.1063/1.4979676 PMC5533476 28798849

[51] Sackmann EK, Fulton AL, Beebe DJ (2014). The present and future role of microfluidics in biomedical research. Nature..

